# Structural and Functional Characterization of Three Novel Fungal Amylases with Enhanced Stability and pH Tolerance

**DOI:** 10.3390/ijms20194902

**Published:** 2019-10-03

**Authors:** Christian Roth, Olga V. Moroz, Johan P. Turkenburg, Elena Blagova, Jitka Waterman, Antonio Ariza, Li Ming, Sun Tianqi, Carsten Andersen, Gideon J. Davies, Keith S. Wilson

**Affiliations:** 1 York Structural Biology Laboratory, Department of Chemistry, University of York, Heslington, York YO10 5DD, UK; Christian.Roth@mpikg.mpg.de (C.R.); olga.moroz@york.ac.uk (O.V.M.); Johan.turkenburg@york.ac.uk (J.P.T.); lena.blagova@york.ac.uk (E.B.); jitka.waterman@diamond.ac.uk (J.W.); antonio.ariza@path.ox.ac.uk (A.A.); gideon.davies@york.ac.uk (G.J.D.); 2 Carbohydrates: Structure and Function, Biomolecular Systems, Max Planck Institute of Colloids and Interfaces, 14195 Berlin, Germany; 3 Diamond Light Source, Diamond House, Harwell Science and Innovation Campus, Fermi Ave, Didcot OX11 0DE, UK; 4 Sir William Dunn School of Pathology, University of Oxford, Oxford OX1 3RE, UK; 5 Novozymes (China) Investment Co. Ltd., 14 Xinli Road, Haidian District, Beijing 100085, China; MLIX@novozymes.com (L.M.); TQSU@novozymes.com (S.T.); 6 Novozymes (Denmark), Krogshojvej 36, DK-2880 Bagsvaerd, Denmark; CarA@novozymes.com

**Keywords:** α-amylase, starch degradation, biotechnology, structure

## Abstract

Amylases are probably the best studied glycoside hydrolases and have a huge biotechnological value for industrial processes on starch. Multiple amylases from fungi and microbes are currently in use. Whereas bacterial amylases are well suited for many industrial processes due to their high stability, fungal amylases are recognized as safe and are preferred in the food industry, although they lack the pH tolerance and stability of their bacterial counterparts. Here, we describe three amylases, two of which have a broad pH spectrum extending to pH 8 and higher stability well suited for a broad set of industrial applications. These enzymes have the characteristic GH13 α-amylase fold with a central (β/α)_8_-domain, an insertion domain with the canonical calcium binding site and a C-terminal β-sandwich domain. The active site was identified based on the binding of the inhibitor acarbose in form of a transglycosylation product, in the amylases from *Thamnidium elegans* and *Cordyceps farinosa*. The three amylases have shortened loops flanking the nonreducing end of the substrate binding cleft, creating a more open crevice. Moreover, a potential novel binding site in the C-terminal domain of the *Cordyceps* enzyme was identified, which might be part of a starch interaction site. In addition, *Cordyceps farinosa* amylase presented a successful example of using the microseed matrix screening technique to significantly speed-up crystallization.

## 1. Introduction

The use of enzymes in industrial processes is a multi-billion-dollar market. One of the first enzymes discovered in 1833 was diastase, an enzyme able to hydrolyze starch [1]. Nowadays, amylases, also able to hydrolyze starch, constitute up to 25% of the market for enzymes and have virtually replaced chemical methods for degrading starch in the industrial sector (reviewed in [2]). Amylases are the most important class of enzymes for degrading starch and can be subdivided into three subclasses: α-, β-, and gluco-amylases based on their reaction specificity and product profiles. α-amylases degrade the α- 1,4 linkage between adjacent glucose units and are extensively used for example in bioethanol production or in washing powder and detergents [3] (and reviewed in [4]). One of the most widely used α-amylases is that from *Bacillus licheniformis*, known under the tradename “Termamyl”. Microbial amylases are generally used in detergent applications and other industrial processes, including bioethanol production, with new amylases, in particular those from hyperthermophilic organisms, offering further improvement in the production process (reviewed in [5]).

α-amylases belong to glycoside hydrolase family 13 (GH13) in the CAZy database classification [6]. They have a (β/α)_8_ barrel domain harboring the active site, a subdomain which includes the canonical calcium binding site inserted between the third β-strand and the third α-helix and a C-terminal β-sandwich domain, thought to be important for the interaction with raw starch (reviewed in [7]) [8,9]. Amylases follow a retaining mechanism with an aspartate as nucleophile and one glutamate as general acid/base [10,11]. Up to ten consecutive sugar subsites forming the active site cleft have been identified in bacterial amylases [12]. 

To date, recombinant fungal amylases have been isolated from mesophilic hosts such as *Aspergillus oryzae* and are of particular interest to the food industry as they match the temperature and pH range used in typical applications in the baking process, where they are active in the dough but inactivated during baking. Due to the widespread use of fungal enzymes for the production of food and food ingredients (such as citric acid), they are classified as GRAS (generally recognized as safe) organisms by organizations including the FDA (US Food and Drug Administration) [13].

Up till now, fungal enzymes with a higher pH-tolerance and thermostability have not been reported. Here, we describe the structure and function of three novel α-amylases from *Cordyceps farinosa* (CfAM), *Rhizomucor pusillus* (RpAM) and *Thamnidium elegans* (TeAM) with a higher stability and pH-tolerance with the potential to act as novel biocatalysts for various industrial processes. The sequence of all three enzymes groups them in the GH13 sub-family 1 along with, for example, the amylase from *Aspergillus oryzae* (also known as TAKA amylase). However, unlike other fungal amylases, the enzymes in this study have been shown to have a broad pH profile with an optimum around pH 5 while retaining activity at pH 8. Furthermore, their more open crevice leads to the production of longer oligomers compared to TAKA amylase.

The native RpAM and TeAM have a four-domain fold with a carbohydrate binding domain (CBM20) at the C-terminus and a short serine-rich linker in between, while native CfAM lacks this CBM20 domain. In this study, only the core of the amylases including the A, B and C domains was cloned and expressed. In addition, crystallization of *Cordyceps farinosa* amylase again demonstrates the power of the microseed matrix screening technique [14].

## 2. Results

### 2.1. Biochemical Characterization

**Figure 1:**
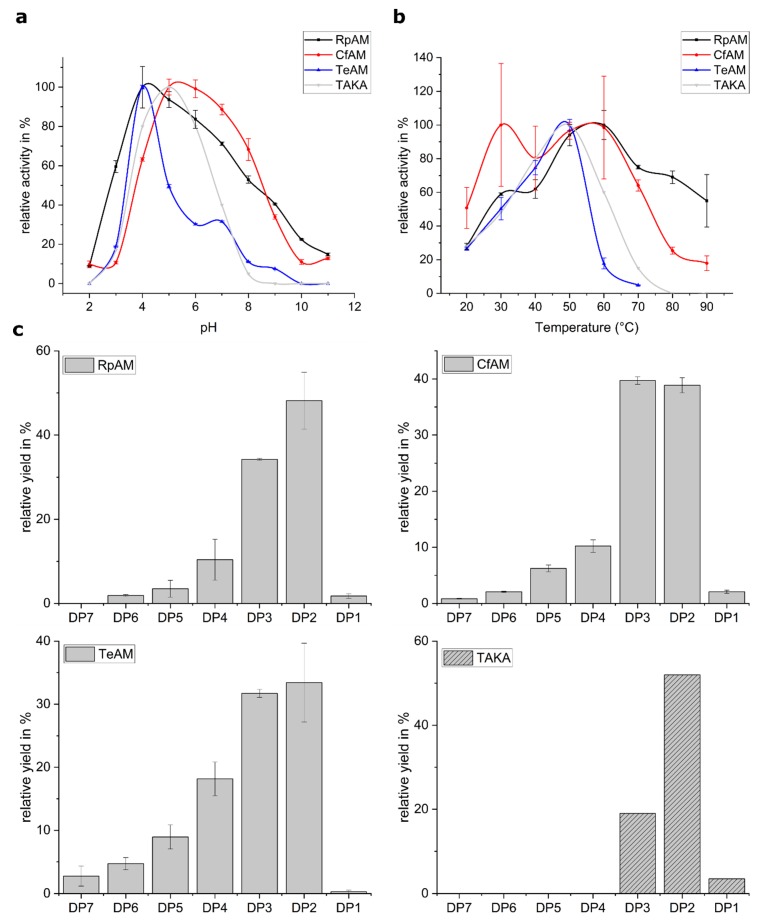
Biochemical characterization of RpAM, CfAM. TeAM and TAKA. (**a**) pH-profile of all three amylases in comparison with TAKA amylase; (**b**) temperature profile of all three amylases in comparison with TAKA amylase; (**c**) product profile of all three amylases and the abundance of oligomers with a degree of polymerization (dp) of 1 to 7 after hydrolysis of starch.

The pH, temperature and product profiles were characterized for all three amylases. Of great desire are amylases with a broader pH-tolerance compared to TAKA amylase. Our analysis showed that all three amylases have a pH optimum around 5. Whereas TeAM has no significant activity above pH 7, RpAM and CfAM retain significant activity at pH 7 extending up to a pH of 9 (Figure 1a). In particular, CfAM shows the highest pH tolerance, retaining 70% of its activity at pH 8. RpAM and TeAM both show a pronounced shoulder, suggesting the involvement of more titratable residues in the substrate recognition and catalysis process. The temperature profiles reveal that RpAM and CfAM also have a considerably higher thermotolerance compared to TAKA and TeAM (Figure 1b). In particular, RpAM retains full activity even at 80 °C, making it an attractive enzyme for industrial high temperature starch saccharification processes. Compared to TAKA amylase, all three amylases show a tendency to produce higher amounts of oligomers with a degree of polymerization (dp) of three, with trace amounts of oligomers with a dp of up to seven for TeAM (Figure 1c). 

### 2.2. Overall Fold

**Figure 2:**
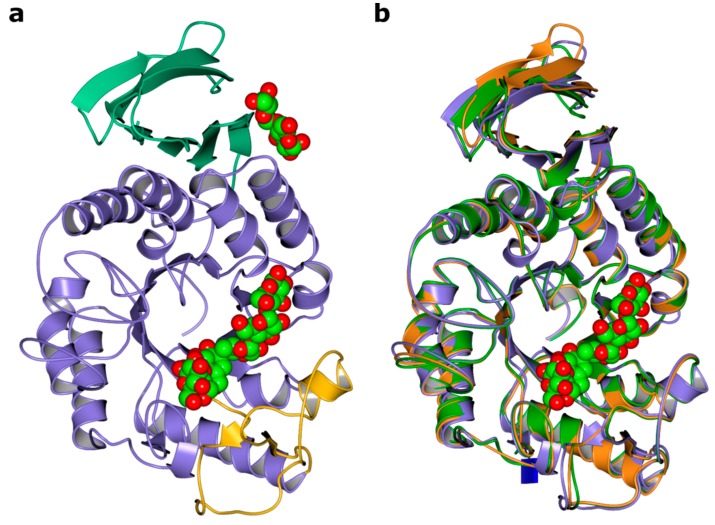
Structural overviews. (**a**) ribbon representation of the structure of CfAM amylase in ribbon representation. The domains are colored separately with the central barrel in purple. subdomain B in yellow and the C-terminal β-sandwich in green. The bound ligands acarbose transglycosylation product (ATgp) and maltose are shown as spheres; (**b**) structural superposition of CfAM (purple) TeAM (orange) and RpAM (green).

The structures were solved using molecular replacement starting from the *A. oryzae* amylase as template (pdb-ID: 7taa and 3vx0) to a resolution of 1.4 Å for RpAM, 1.2 Å for TeAM and 1.35 Å for CfAM, respectively. The final model of RpAM includes two monomers in the asymmetric unit comprising residues 1 to 438 in both chains, which superpose on each other with an r.m.s.d. of 0.54 Å. The model of TeAM contains one monomer in the asymmetric unit including residues 1 to 438. For CfAM, there are two monomers in the asymmetric unit comprising residues 19 to 459 for chain A and 19 to 460 for chain B, which superpose with an r.m.s.d. of 0.3 Å. All three amylases have the classical domain structure with a central (β/α)_8-_barrel with the active site located on its C-terminal face, together with a small subdomain, inserted between the third strand and helix and a C-terminal β-sandwich (Figure 2a). All three superpose with each other (Figure 2b) and with TAKA-amylase with an r.m.s.d. between 0.6 to 0.9 Å for up to 423 residues. Two conserved disulphide bridges stabilize flexible loops in subdomains A and B. There is an additional disulphide bridge in CfAM, located in the C-terminal domain. All three α-amylases have the conserved canonical calcium binding site located between the (β/α)_8-_barrel and the insertion domain B. 

### 2.3. Ligand Binding Site

**Figure 3:**
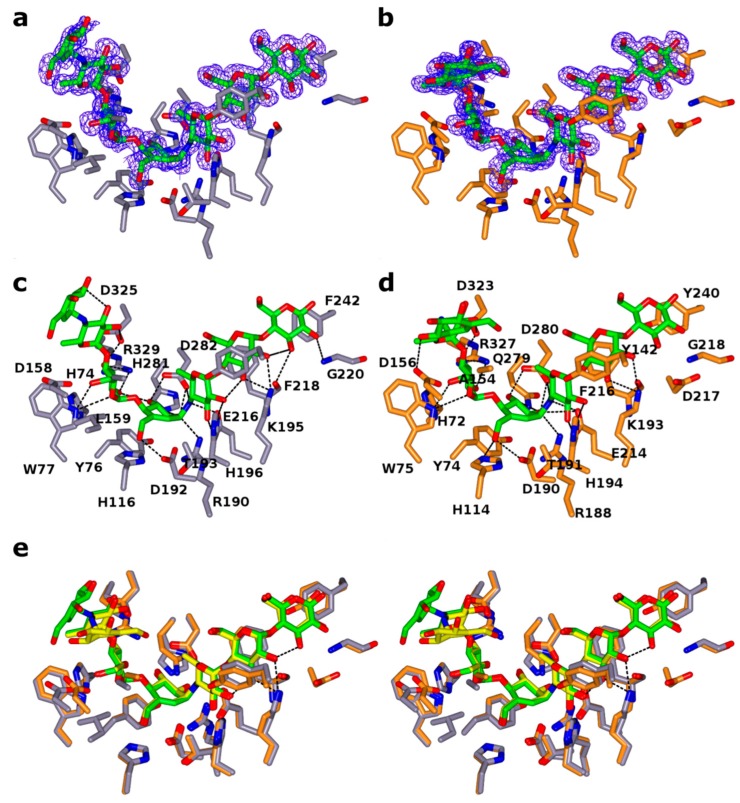
Acarbose transglycosylation product binding in CfAM and TeAM. (**a**,**b**) stick representation of the acarbose derived transglycosylation product in the substrate binding crevice of CfAM and TeAM, respectively. The 2Fo-Fc electron density around the ligands is contoured at 0.3 e/Å^3^. The interacting residues are shown as cylinders. (**c**,**d**) hydrogen bonding pattern between ATgp and CfAM and TeAM in the active site. (**e**) stereo view of the overlay of the binding crevice of CfAM (purple) and TeAM (orange). The residues and the ligands overlap very closely with the only major difference being the orientation of the acarviosine subunit in subsite -4.

Although all three amylases were co-crystallized with acarbose, a well-known inhibitor for amylases, a complex with acarbose bound was only obtained for TeAM and CfAM. The reason why acarbose was not bound to RpAM is not clear. As expected, the acarbose was found in the substrate binding cleft in each monomer of TeAM and CfAM, with the acarviosine unit sitting in subsites -1 and +1, (Figure 3a–d). In both enzymes, the binding mode is conserved, and the ligands superpose with each other (Figure 3e), except for the monomer in subsite -4. The distorted pseudosugar valieneamine in subsite 1 with its ^2^H_3_ half chair conformation mimics the conformation of the putative transition state along the catalytic itinerary of α-amylases. Additional density in subsites -2 and -3 and -4 was modelled as a second acarbose unit, covalently attached to the first acarbose. The catalytic nucleophile D190/D192(CfAM/TeAM) is in a near attack conformation poised to react with the anomeric carbon, whilst the catalytic acid/base E214/E216(CfAM/TeAM) forms a hydrogen bond with the bridging nitrogen of the glycosidic bond with the 4-deoxyglucose in subsite +1. In addition, a hydrogen bond with H194/H196 stabilizes the 4-deoxyglucose in that subsite. The +3 subsite is formed by the sugar tong, composed of Y142/144 of subdomain B and F216/218 of the central domain, sandwiching the glucose between them. The reducing end of acarbose is stabilized by a hydrophobic platform interaction with Y240/F242 and a hydrogen bond with the main chain nitrogen of G218/G220. Interestingly, additional density at the non-reducing end was observed and was modelled as an additional acarbose unit in subsites −2 and −3 and −4. The glucose in subsite −2 is stabilized by multiple hydrogen bonds with D323/325, R327/329 and W375/377. The glucose in subsite −3 is held in place by only one hydrogen bond with D323/325. The last visible part of the acarbose molecule is the acarviosine unit in subsite −4, which is not stabilized by direct interactions with the protein. Furthermore, the acarviosine unit is in two different positions in the two structures, reflecting the lack of strong stabilizing interactions between the ligand and the protein beyond subsite −3 (Figure 3e). 

### 2.4. Secondary Glucose Binding Site

**Figure 4:**
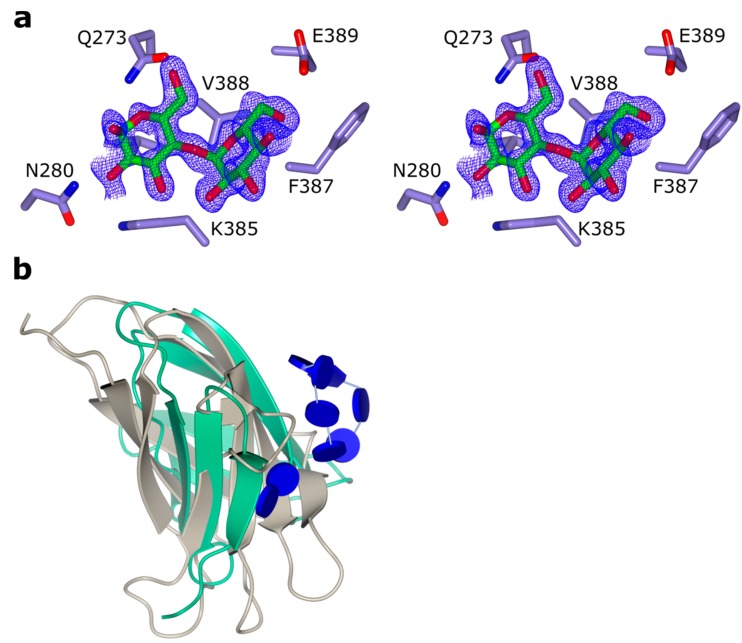
The secondary maltose binding site in the C-terminal domain of CfAM. (**a**) stereo view showing the maltose in cylinder representation with the corresponding 2Fo-Fc electron density contoured at 0.4 e/Å^3^. The interacting residues are shown as blue cylinders; (**b**) superposition of the C-terminal domain (green) with the CBM20 domain from *A. niger* glucoamylase (pdb-ID: 1ac0) in beige. The bound β-cyclodextrin of CBM20 and the maltose unit are shown as glycoblocks [15].

In CfAM, a secondary binding site in domain C was identified and modelled as maltose located at the edge of the β-sandwich (Figure 4). The glucose units are held in place mainly via hydrogen bonds without the usual stacking interactions with aromatic side chains. 

### 2.5. N-Glycosylation

There are three N-glycosylation sites, one at N144 in RpAM and two at N180 and 412 in TeAM. We observed only the core GlcNAc residue in all three enzymes. In the case of TeAM, this is due to the deglycosylation procedure with EndoH. 

### 2.6. Isoasparate Formation

We observed the formation of an isoaspartate by succinimide formation and deamidation of N120 in chain B of RpAM. The same asparagine in chain A shows high flexibility and the resulting density suggest partial isoaspartate formation, but a model could not be built with confidence. 

## 3. Discussion

We have analyzed structurally and functionally three novel fungal α-amylases with potential to be used in the food industry and other industrial processes. All three structures determined show the canonical amylase fold and overlap with each other with an r.m.s.d. of 0.54 Å (Figure 1b). Further analysis of the sequence showed that both RpAM and CfAM have a slightly lower number of charged residues and a higher number of hydrophobic residues compared to TeAm and TAKA amylase, which might contribute to the higher thermostability of these two variants. Increased internal hydrophobicity while keeping external hydrophilicity was found to correlate well with the thermostability of *Bacillus* α-amylases [16]. Furthermore, the shortened loops in these enzymes may also contribute to the overall rigidity of the enzymes and therefore the thermostability as observed for other enzymes as well [17,18]. 

The substrate crevice in all three amylases, if defined on the basis of protein carbohydrate interactions, spans from subsite -3 to +3. Having only three defined subsites for the non-reducing end is common for amylases and is in line with the number of donor subsites described for the TAKA-amylase. Potentially, there could be more subsites for additional carbohydrate units at the reducing end, which might connect the active site crevice with the observed second binding site (see below).

The observed complexes are most likely the result of limited transglycosylation, an unusual side reaction previously reported *in crystallo* for several amylases—for example, TAKA-amylase [19]. Though this reaction is common in the closely related CGTases (GH13_2) and amylomaltases (GH77), it was not observed in solution for α-amylases. However, in crystals, transglycosylation products with 10 or more units have been reported as a result of multiple transglycosylation events. Interestingly, the final complex always has the pseudosaccharide unit, thought to mimic the transition state, in the -1 subsite, rendering the enzyme inactive. Other binding modes are clearly possible as evidenced by the final product and a pre-Michaelis complex observed for GH77 *Thermus aquaticus* amylomaltase with acarbose [20]. 

**Figure 5:**
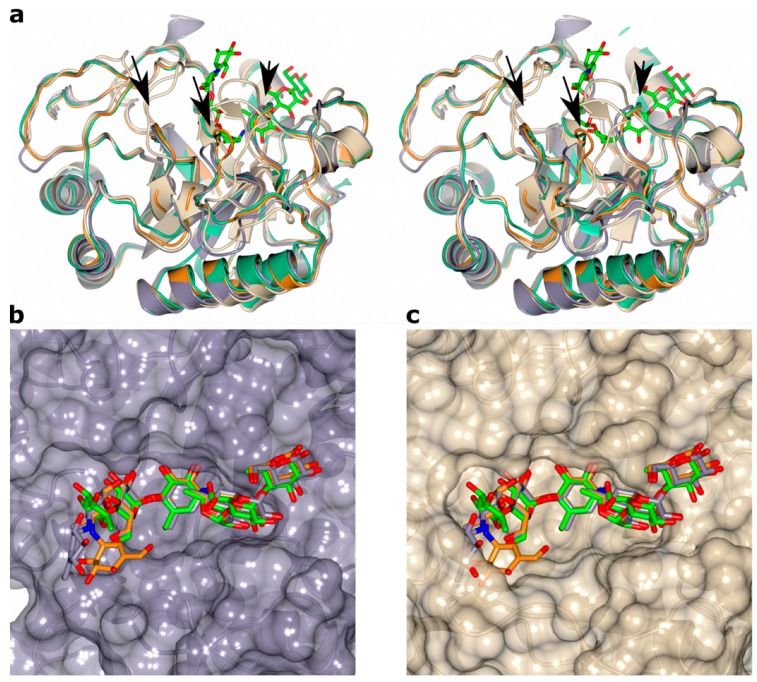
(**a**) Stereo view of all three amylases compared to TAKA-amylase with the three shortened loops in the front marked with arrows. The ligand in CfAM is shown as sticks to identify the active site; (**b**) surface representation of CfAM with the bound ligand. The substrate is more open on the donor subsite; (**c**) surface representation of TAKA-amylase. The elongated loops create a more restricted active site crevice precluding the binding mode observed in CfAM and TeAM due to steric clashes.

All three amylases have as their hallmark a shortened loop between β2/α3 and two shorter loops in subdomain B located between β3 and α4 of the central (β/α)_8_-barrel, compared to structures of other fungal amylases, e.g., TAKA-amylase (Figure 5a). The importance of subdomain B for the physicochemical properties—for example, pH-stability, as well as substrate and product specificity, is well known [21,22,23,24]. Indeed, the shorter loops open up the substrate crevice on the non-reducing end (Figure 5b), which might explain the shift in the product profile for all three amylases towards oligomers with a higher dp compared to TAKA amylase (Figure 1c and Figure 5c). 

The C-terminal domain in α-amylases is implicated in starch binding and shows structural similarity to classic CBM domains, based on an analysis using PDBeFOLD [25]. The additional binding site in this domain in CfAM strengthen the role of this domain in substrate binding. Additional carbohydrate binding sites have been observed as well for example in barley α-amylase 1 [26]. While none of these sites overlap with the binding site seen in CfAM, a structure of a CBM20 in complex with β-cyclodextrin revealed two binding sites, with the site termed SB1 in close proximity to the binding site in CfAM (Figure 4b) [27]. This was confirmed to be the primary binding site for the interaction with raw starch, and it is likely that the observed binding site in CfAM is a genuine carbohydrate binding site. Furthermore, it is intriguing to speculate about a potential path from the primary substrate crevice to the secondary glucose binding site, which could be rather easily thought as a simple extension of the acarbose from the reducing end. 

Only limited information about the influence of glycosylation on amylase activity is available. It was shown that, for α-amylase, Amy1 from the yeast *Cryptococcus flavus* N-glycosylation enhances thermostability and resistance to proteolytic degradation [28]. The same effect is observed for *Trichoderma reesei* Cel7a [29]. Indeed, N144 is located in an extended loop and N-glycosylation might help to shield the loop against proteolytic attack. The other two glycosylation sites are located in or at the beginning of secondary structure elements, with N412 being located in the C- domain. 

The observed isoaspartate formation is thought usually to be an age-related side effect of protein decomposition, but a functional role cannot be ruled out [30]. Indeed, it was shown in GH77 enzymes that such unusual posttranslational rearrangement might play a functional role in glycoside hydrolases [31,32]. The observed isoaspartate is located in one of the shortened loops in subdomain B close to the substrate binding cleft, suggesting a functional role in CfAM as well. 

## 4. Materials and Methods 

### 4.1. Macromolecule Production 

The coding sequence of CfAM for the A, B and C domains was amplified from *Cordyceps farinosa* gDNA by the polymerase chain reaction (PCR). The PCR fragment was obtained using primer pairs: 5′-ACACAACTGGGGATCCACCATGAAGCTTACTGCGTCCCTC-3′ and 5′-GATGGTGATGGGATCCTTACTGCGCAACAAAAACAATGGG-3′. The fragment was then ligated in the expression vector pSUN515 using *Bam*HI and *Xho*I restriction sites. The ligation protocol was performed according to the IN-FUSION™ Cloning Kit instructions. A transformation of TOP10 competent *E. coli* cells (Tiangen, Beijing China) with the plasmid, containing the CfAM gene, was performed and positive clones confirmed by sequencing. The transformation of *Aspergillus oryzae* (strain *MT3568*) with the expression vector comprising CfAM gene was performed according to patent application WO95/002043 [33]. After incubation for 4–7 days at 37 °C, spores of four transformants were inoculated into 3 mL of YPM medium. After 3-day cultivation at 30 °C, the culture broths were analyzed by SDS-PAGE to identify the transformant producing the largest amount of recombinant mature amylase with an estimated size of 48 kDa. Spores from the best expressing transformant were cultivated in YPM medium in shake flasks for 4 days at a temperature of 30 °C. The culture broth was harvested by filtration using a 0.2 µm filter device, and the filtered fermentation broth was used for purification and further assays.

RpAM was cloned and expressed in a similar manner as CfAM while TeAM was expressed in *Pichia pastoris* with a similar protocol to that described for the lipase from *Gibberella zeae* [34]. The entire coding sequence of TeAM was amplified from cDNA by the polymerase chain reaction and transformation into ElectroMax DH10B competent cells (Invitrogen, Waltham, MA, USA) by electroporation. Transformed cells were plated on LB plates containing 100 mM ampicillin. After overnight incubation at 27 °C, a positive clone was selected by colony PCR and confirmed by sequencing. The plasmid DNA of the positive clone was linearized with PmeI (NEB, Ipswich, MA, USA) and transformed into *Pichia pastoris* KM71 (Invitrogen, Waltham, MA, USA) following the manufacturer’s instructions. An amylase positive clone was inoculated into 3 mL buffered minimal sorbitol complex medium and incubated at 28 °C for 3 days until the OD600 reached 20. Methanol was added to the culture daily to a final concentration of 0.5% for the following 4 days. On day 4 of induction, the culture supernatant was separated from the cells by centrifugation and the pH of the supernatant was adjusted to 7.0.

The CfAM culture broth was precipitated with ammonium sulphate (80% saturation), then dialyzed with 20 mM Na-acetate at pH 5.0. The solution was loaded on to a Q Sepharose Fast Flow column (GE Healthcare, Brondby, Denmark) equilibrated with 20 mM Na Acetate at pH 5.0. Protein was eluted with a salt gradient from zero to 1 M NaCl Fractions were analyzed for amylase activity and pooled accordingly. The flow-through fraction, containing the bulk of amylase activity was supplemented with ammonium sulphate to a final concentration of 1.2 M and then loaded on to Phenyl Sepharose 6 Fast Flow column (GE Healthcare, Brondby, Denmark). The activity was eluted by a linear gradient of decreasing salt concentration. The fractions with activity were analyzed by SDS-PAGE and then concentrated for further use.

Amylase activity was detected by Azo dyed and azurine cross-linked hydroxyethyl-amylose (AZCL-HE-amylose) (Megazyme International Ireland Ltd., Bray, Ireland) as substrate. In addition, 10µL enzyme sample and 120 µL 0.1% substrate at pH 7 were mixed in a microtiter plate and incubated at 50 °C for 30 min. Then, 70 µL supernatant was transferred to a new microtiter plate and the absorption at 595 nm determined. All reactions were done as duplicates.

### 4.2. Biochemical Characterisation

#### 4.2.1. pH Optimum

To determine the pH Optimum, each enzyme (3 µL of a 0.5 mg/mL solution) was incubated with 40 µL 1% substrate (AZCL-HE-amylose) (Megazyme International Ireland Ltd., Bray, Ireland). The pH between 2 and 11 was adjusted using 100 µL of B&R buffer (Britton–Robinson buffer: 0.1 M boric acid, 0.1 M acetic acid, and 0.1 M phosphoric acid, adjusted to pH-values 3.0, 4.0, 5.0, 6.0, 7.0, 8.0, 9.0, 10.0 and 11.0 with HCl or NaOH) [35]. The reactions were incubated at 30 °C for 30 min and afterwards 60 µL were transferred in a new microtiter plate and the absorption was measured at 595 nm. 

#### 4.2.2. Temperature Optimum

To determine the Temperature Optimum, each enzyme was incubated with 100 µL 0.1% substrate (AZCL-HE-amylose) (Megazyme International Ireland Ltd., Bray, Ireland) in 50 mM Na Acetate pH 4.3. The substrate solution was preincubated at 20–90 °C for 5 min and the reaction was started by addition of 3 µL of enzyme solution (0.5 mg/mL). The reaction mixture was further incubated at the respective temperature for 30 min at 950 rpm. The reaction was stopped by rapid cooling on ice. Afterwards, 60 µL of each reaction was transferred in a microtiterplate and the absorption was measured at 595 nm. Each reaction was performed in triplicate. 

#### 4.2.3. Product Profile

For product profile determination, each enzyme (15 µL) was incubated with 120 µL 0.1% substrate (AZCL-HE-amylose) (Megazyme International Ireland Ltd., Bray, Ireland) at pH 5 and 62 °C for 14 h. 70 µL of each reaction was mixed with equal amounts of Acetonitril. The mixture was centrifuged for 30 min at 16.000× *g* and the supernatant was analyzed using HPAEC with pulsed amperometric detection.

### 4.3. Crystallisation

#### 4.3.1. RpAM

The concentrated protein was mixed with acarbose in a molar ration of 4:1 before the initial screening in 96 well format using commercially available screens. An initial hit (0.2 M NaCl, 0.1 M Na-acetate pH 4.6, 30% MPD) was further refined in 24-well format using the initial crystals as seeds. Crystals suitable for data collection were cryoprotected using 25% glycerol and flash frozen in liquid nitrogen prior data collection. 

#### 4.3.2. TeAM

Prior to providing the sample to York, the protein was deglycosylated using Endo-H treatment. The protein was concentrated using Amicon (Merck, Germany) filter units and stored at −80 °C for later use. For the crystallization, the protein was mixed with 5 mM acarbose prior to setting up the screen. Initial screens were set up in a 96-well sitting drop format using commercially available screens. Initial hits were further refined in a 24-well hanging drop format. The best crystals grew in 0.1 M di-hydrogen phosphate, 1.8 M ammonium sulphate. Crystals were cryoprotected by addition of ethylene glycol to a final concentration of 15%. The crystals were flash frozen in liquid nitrogen prior to data collection.

#### 4.3.3. CfAM

**Figure 6:**
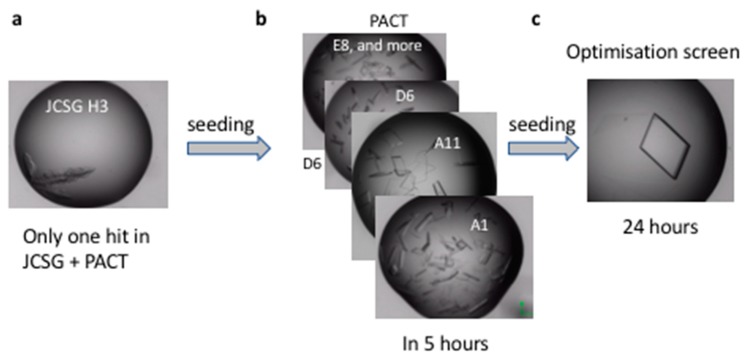
Crystal optimization using microseed matrix screening.

Prior to crystallization, the protein was concentrated to 22.5 mg/mL by ultrafiltration in an Amicon centrifugation filter unit (Millipore), aliquoted to 50 µL; aliquots that were not immediately set up for crystallization were flash frozen in liquid nitrogen and stored at ™80°C to use later in optimizations. Initial crystallization experiments were carried out in the presence or absence of 4 mM CaCl_2_ and 40 mM acarbose. An initial hit was obtained for an acarbose complex, in just one condition (H3, Bis-tris 5.5, 25% w/v PEG3350) of JCSG screen (Figure 6a), out of total 192 conditions in two initial screens set up – JCSG and PACT premier™ HT-96 (Molecular Dimensions (Suffolk, UK)). The crystals were imperfect and were used to make the seeding stock. The seeding stock was prepared and microseed matrix screening (MMS, recent review in [14]) carried out using an Oryx robot (Douglas Instruments (Hungerford, UK)) according to the published protocols [36,37]. Briefly, crystals were crushed, and diluted with ~50 µL of mother liquor. The solution was transferred into a seed bead containing reaction tube and vortexed for three minutes. The seeding stock was used straightaway, and the remaining seeds were frozen and kept at ™20 °C. MMS was carried out in the PACT screen, giving a significant number of hits (Figure 6b). Crystals from condition A11 were used to make a seeding stock for the next seeding round. This time it was not a “classical” MMS-seeding into a random screen, but rather seeding into an optimization screen based on the initial conditions, but with different pH, salts and PEGs/PEG concentrations. The crystallization drops contained 150 nl protein + 50 nl seeding stock + 100 nl mother liquor from a new random screen. The final, good quality crystal was obtained in 12% PEG 3350 0.2 M NaNO_3_, CAPS pH 11.0 (Figure 6c).

### 4.4. Data Collection and Processing

The data were collected at Diamond on beam line I02, processed by XDS [38], and scaled with Aimless [39]. The statistics are shown in Table 1.

### 4.5. Structure Solution and Refinement

The structure of RpAM was solved by molecular replacement with Molrep [40], using TAKA amylase as template (PDB-ID:7taa). The structure of TeAM was solved with Molrep using the model of RpAM. The CfAM structure was solved using Molrep [40] with 3vx0 α-amylase from *Aspergillus oryzae* as a model. The final models were built using automated chain tracing with Buccaneer [41], followed by manual building in Coot [42], iterated with reciprocal space refinement using Refmac [43]. The statistics are summarized in Table 2.

## 5. Conclusions

Taken together, we describe the structural and functional characterization of three novel fungal α- amylases with enhanced stability, of which two, CfAM and RpAM, have a higher pH optimum and greater temperature tolerance, well suited for usage in the detergent or saccharification industry. The structures reveal that these amylases follow the canonical domain structure of α-amylases, and that three shortened loops between β_2_/α_3_ and in subdomain B are likely to be responsible for the altered enzymatic properties of the amylases compared to TAKA-amylase. For the first time, we have unambiguously identified up to three different N-glycosylation sites in α-amylases in the structures. Furthermore, the observed formation of an isoaspartate from an asparagine in one of the shortened loops might play a functional role. The complexes with acarbose derived transglycosylation products define seven subsites of the substrate binding crevice and helped to identify the catalytic residues unambiguously. In addition, a new previously unobserved carbohydrate binding site was revealed in the C-terminal β-sandwich domain of CfAM, which might be important for the initial interaction with its polymeric substrate.

## 6. Patents

The *Rhizomucor pusillus* amylase and the use of this amylase in various industrial applications have been claimed in patent application WO2006065579. A close homologue of the *Thamnidium elegans* amylase was claimed in patent application WO2006069290 including the use in industrial applications.

## Figures and Tables

**Table 1:** Data collection and processing statistics.

	CfAM	TeAM	RpAM
Diffraction source	Diamond I02	Diamond I02	ESRF ID29
Wavelength (Å)	0.9795	0.9795	1.0004
Temperature (K)	100	100	100
Space group	P1	P2_1_2_1_2_1_	P1
*a*, *b*, *c* (Å)	56.88, 61.97, 70.40	51.02, 56.63, 166.01	51.22, 62.60, 66.81
α, β, γ (°)	79.33, 82.88, 67.99	90, 90, 90	77.03, 81.04, 89.62
Resolution range (Å)	33.1–1.35 (1.37–1.35)	48.76–1.20 (1.22-1.20)	43.21–1.4 (1.42–1.40)
Total No. of reflections	342,708	1,149,540	315,876
No. of unique reflections	163,777	150,529	146,177
Completeness (%)	85.3 (38.2)	99.8 (96.7)	92.9 (61.9)
Redundancy	2.1 (2.1)	7.6 (4.6)	2.2 (2.1)
〈*I*/σ(*I*)〉	13.7 (10.3)	14.1(1.7)	9.6 (2.3)
*R*_r.i.m._	0.076 (0.129)	0.021 (0.446)	0.030 (0.225)
*CC1/2*	0.983 (0.970	0.999 (0.615)	0.998 (0.892)
Overall *B* factor from Wilson plot (Å^2^)	6.8	8.7	8.1
			

Values for the outer shell are given in parentheses.

**Table 2:** Structure solution and refinement.

	CfAM	TeAM	RpAM
PDB-ID	6SAV	6SAO	6SAU
Resolution range (Å)	33.1–1.35 (1.385–1.35)	48.76–1.20 (1.22–1.20)	39.99–1.4 (1.42–1.40)
Completeness (%)	85.3 (39.7)	99.8 (96.7)	92.8 (89.2)
No. of reflections, working set	155,488	143,033	138,848
No. of reflections, test set	8289	7574	7328
Final *R*_cryst_	0.113 (0.09)	0.110 (0.27)	0.136 (0.22)
Final *R*_free_	0.150 (0.17)	0.134 (0.29)	0.164 (0.26)
Cruickshank DPI	0.051	0.027	0.056
No. of subunits in the asymmetric unit	2	1	2
No. of non-H atoms	Chain A/B	Chain A	Chain A/B
Protein	3557/3609	3570	3662/3592
Ion	1/2	1	1/1
Ligand	99/120	133	14/36
Water	875	568	943
Total	8263	4272	8306
R.m.s. deviations			
Bonds (Å)	0.0191	0.0163	0.0147
Angles (°)	2.06	1.937	1.875
Average *B* factors (Å^2^)	Chain A/B	Chain A	Chain A/B
Protein	10/8.7	12.9	12.9/12.3
Ions			
Ca^2+^	6.7/5.8	9.5	7.39/7.5
Na^2+^	N/A/10.9		
Ligand	19.6/18.0	22.2	19.2/21.4
Water	19.0	28.8	24.21
Ramachandran plot			
Most favoured (%)	98.6	97.7	97.2
Allowed (%)	1.4	2.3	2.7

Values for the outer shell are given in parentheses.

## Abbreviations

CfAM.	*Cordyceps farinosa* amylase
RpAM	*Rhizomucor pusillus* amylase
TeAM	*Thamnidium elegans* amylase
TAKA	*Aspergillus oryzae* amylase
dp	Degree of polymerization
